# Accuracy of a zero-heat-flux thermometer in cardiac surgery, a prospective, multicentre, method comparison study

**DOI:** 10.1038/s41598-024-53647-3

**Published:** 2024-02-07

**Authors:** Carsten Pedersen, Peter Munch, Jesper Kjaergaard, Lars Grønlykke, Anselm Bräuer

**Affiliations:** 1 Department of Cardiothoracic Anesthesiology, Copenhagen University Hospital, 2100 Copenhagen, Denmark; 2 Department of Cardiothoracic Anesthesiology, Aalborg University Hospital, Aalborg, Denmark; 3 Department of Cardiology, The Heart Centre, Copenhagen University Hospital, Copenhagen, Denmark; 4 Department of Anesthesiology, University Hospital Göttingen, Göttingen, Germany

**Keywords:** Cardiology, Health care, Risk factors, Fever

## Abstract

Accurate measurement of core temperature is of utmost importance during on-pump cardiac surgery, for detection of hypothermia before cardiopulmonary bypass (CPB), guidance of temperature management on CPB, active rewarming on CPB and guidance of warming therapy after CPB. Most temperature measurement methods are known to become inaccurate during rapid changes in core temperature and suffer from delayed detection of temperature changes. Zero-heat-flux temperature (ZHF) measurement from the lateral forehead may be an alternative, non-invasive method quantifying the core temperature. A prospective, observational, multicentre study was conducted in one hundred patients scheduled for on-pump coronary artery bypass grafting. Core temperatures were measured every minute by two zero-heat-flux thermometer (SpotOn™) and a bladder thermometer and a pulmonary artery catheter (PAC) in the period after induction of anesthesia until CPB. Accuracy and precision of both methods were compared against core temperature measured in the pulmonary artery using the method of Bland and Altman. A high accuracy (around 0.1 °C) and a very good precision (Limits of agreement (LoA) − 0.6; 0.4 °C) were found between zero-heat-flux thermometer and core temperature measured by PAC. Among the two ZHF thermometers the bias was negligible (− 0.003 °C) with narrow LoA of − 0.42 °C and 0.41 °C. In contrast, bias between bladder temperature and PAC temperature was large (0.51 °C) with corresponding LoA of − 0.06 °C and 1.1 °C. ZHF thermometers are in contrast to bladder temperature a reliable core temperature monitor in cardiac surgery during the period after induction of anestesia until CPB. The zero-heat-flux method can provide clinicians reliably with continuous and non-invasive measurements of core temperature in normothermic and mild hypothermic temperature ranges and therefore can be helpful to guide temperature management.

## Introduction

During on-pump cardiac surgery monitoring of core temperature is mainly used for detection of hypothermia before cardiopulmonary bypass (CPB), guidance of temperature management on CPB, active rewarming on CPB and guidance of warming therapy after CPB. Therefore, accurate measurement of core temperature is of utmost importance in these patients.

In general, it is recommended that core temperature should be measured in patients undergoing general anesthesia for more than 30 min^1^, which is always the case in patients undergoing on-pump cardiac surgery. In addition the American Society of Anesthesiologists’ standards for basic anesthetic monitoring state that ‘‘Every patient receiving anesthesia shall have temperature monitored when clinically significant changes in body temperature are intended, anticipated or suspected”^2^. This statement was reaffirmed in December 2020 and is especially valid for patients undergoing on-pump cardiac surgery.

Core temperature is commonly measured in the mouth, nasopharynx or bladder, and these temperatures provide a reasonably accurate estimation of the core temperature if no extreme thermal perturbations occur^3^. However, most of these measurements are known to become inaccurate during rapid changes in core temperature and suffer from delayed detection of temperature changes. Zero-heat-flux temperature (ZHF) measurement from the lateral forehead may be an alternative, non-invasive method quantifying the core temperature. The measurement range of this method is 25 °C to 43 °C.

To a certain level, noninvasive measurement methods often depend on skin perfusion and vasomotor status. Hypothermia results in peripheral arteriovenous shunt vasoconstriction and thus decreased skin perfusion. In contrast, hyperthermia results in peripheral vasodilatation and increased skin perfusion^3^. This may affect the accuracy of these methods.

The most accurate method to measure core temperature is the measurement of temperature in the pulmonary artery as this temperature represents the temperature of the central blood. However, due to its invasiveness and price^4^ this method is only chosen when there is an indication for advanced hemodynamic monitoring with a pulmonary artery catheter (PAC). In addition, during CPB the pulmonary artery catheter thermistor may not produce an accurate estimate of core temperature because pulmonary blood flow is reduced to a minimum and the proximal pulmonary artery is exposed.

In this study, we aimed primary to compare the core temperature measured with two noninvasive ZHF thermometers and a urinary bladder thermometer against a gold standard blood temperature measured in the pulmonary artery in patients undergoing on-pump cardiac surgery. Secondary we intended to compare the reproducibility of the ZHF measurements by using two devices at the same time in each patient. Our hypothesis was that there is a clinically acceptable agreement between pulmonary artery catheter and ZHF measurements.

## Methods

This prospective, observational, multicentre study carried out at Copenhagen University Hospital and Aalborg University Hospital was approved by The Committees on Health Research Ethics in the Capital Region of Denmark on August 27th, 2017 and was registered with the ID 17025426. As the study was completely non-invasive, the Regional Research Ethics Committees for the region of Hovedstaden waived the requirement for informed consent. Protocol registered at ClinicalTrials.gov ID NCT05737147. Data were then collected between September 2018 and October 2020 in accordance with relevant guidelines and regulations.

The inclusion criteria were defined as adult patients of both genders scheduled for cardiac surgery with CPB for either isolated coronary artery bypass grafting (CABG), aortic valve replacement (AVR), mitral valve replacement (MVR) or combined CABG + AVR surgery. In addition, the patients had to have a PAC inserted for advanced hemodynamic monitoring. Exclusion criteria were any interruption of skin on the forehead, planned direct heat application to the forehead, or previous cerebral stroke, that might affect the accuracy of the ZHF device.

Anesthesia was induced with fentanyl, propofol and rocuronium and was maintained with sevoflurane and fentanyl or sevoflurane and remifentanil infusion. Hypotension after induction of anesthesia was treated primarily with phenylephrine or ephedrine and secondarily with norepinephrine infusion.

After induction of anesthesia the PAC (Edwards Lifescience, Thermodilution Paceport Catheter 931F75, 7.5F (2.5 mm) 110 cm) was inserted in the pulmonary artery and connected to a Vigilance 2-monitor. Core temperature at the tip of the PAC was measured every minute and displayed on the monitor. Two SpotOn™ ZHF temperature monitoring probes (3 M, Model 370 Temperature Monitoring System, St. Paul, MN) were fixed on the skin of the left and right side of the forehead above the eyebrow, as described by the manufacturer, before induction of anesthesia. These devices also measured core temperature every minute and displayed the values on two display screens.

This temperature monitoring system uses ZHF thermometry to measure the patient’s core temperature. The temperature monitoring system gently warms the sensor. After an equilibration period of approximately 3 min heat loss from skin-surface to the environment is reduced to zero and a zero-heat-flux condition is established. An isothermal tunnel between the brain and skin surface is thereby created and core temperature can be measured by the device^5^.

In addition, a temperature-sensing indwelling urinary catheter (Covidien™, Mon-a-Therm™ Foly Catheter with temperature sensor 400TM) was placed after induction of anesthesia to allow continuous drainage of urine and continuous measurement of body temperature.

Core temperatures measured by PAC, ZHF-thermometer and bladder thermometer were recorded with an interval of 1 min on a laptop.

In all patients, core temperature was allowed to drop until going on CPB. There was no external warming or infusion warming before CPB. At initiation of CPB measurements were stopped because during CPB core temperatures measured with PAC does not produce an accurate estimate of core temperature.

### Statistics analysis

A clinically acceptable agreement (i.e., 95% limits of agreement) between pulmonary artery catheter and 3 M SpotOn ZHF measurements was predefined to be ± 0.5 °C. This limit was chosen because a normal human circadian temperature variation is at least this large and no randomized trials demonstrate adverse postoperative consequences from temperature differences < 0.5 °C^6^.

To assess the agreement between core temperature measure with the PAC, the SpotOn, and bladder temperature catheter we used the Bland–Altman random-effects approach for data of repeated measures^7^. Bland–Altman plots were used as a primary statistical method to interpretation of accuracy (bias = mean difference methods) and precision (limit of agreement = 1.96 standard deviation).

Additionally, we calculated the proportion of all differences that were within ± 0.5 °C of core temperatures measured with PAC.

## Results

100 eligible patients scheduled for on-pump coronary artery bypass grafting (CABG), Aortic valve replacement (AVR), Mitral valve replacement (MVR) or CABG + AVR were enrolled. 21 patients were included at Copenhagen University Hospital and 79 at Aalborg University Hospital. 83 patients (83%) were male, 17 (17%) were female. The patients were [mean (SD)] 68 ± 8.8 years old, weighed 85 ± 16.9 kg, and were 175 ± 8.2 cm tall resulting in a mean body mass index (BMI) of 27.8 ± 4.9 (Table 1).

**Table 1:** Patient characteristics.

	Number N = 100
Gender (F/M)	17/83
Age in years	68 ± 8.8
Weight in kg	85 ± 16.9
Hight in cm	175 ± 8.2
BMI in kg/m^2^	27.8 ± 4.9
ASA	
III	53
IV	47
Type of surgery	
Coronary artery bypass grafting, on-pump (CABG)	49
Aortic valve replacement (AVR)	33
Mitral valve replacement (MVR)	9
CABG + AVR	9

Quantitative data are reported as mean (SD).

In 100 patients a total of 2224 min were recorded with core temperatures ranging from 37.7 °C to 33.6 °C. All measurements decreased from a normothermic to a hypothermic state (< 36 °C) in the period from induction of anesthesia until commencement of CPB (on average 2.8 °C).

### Bland Altman analysis

**Figure 1:**
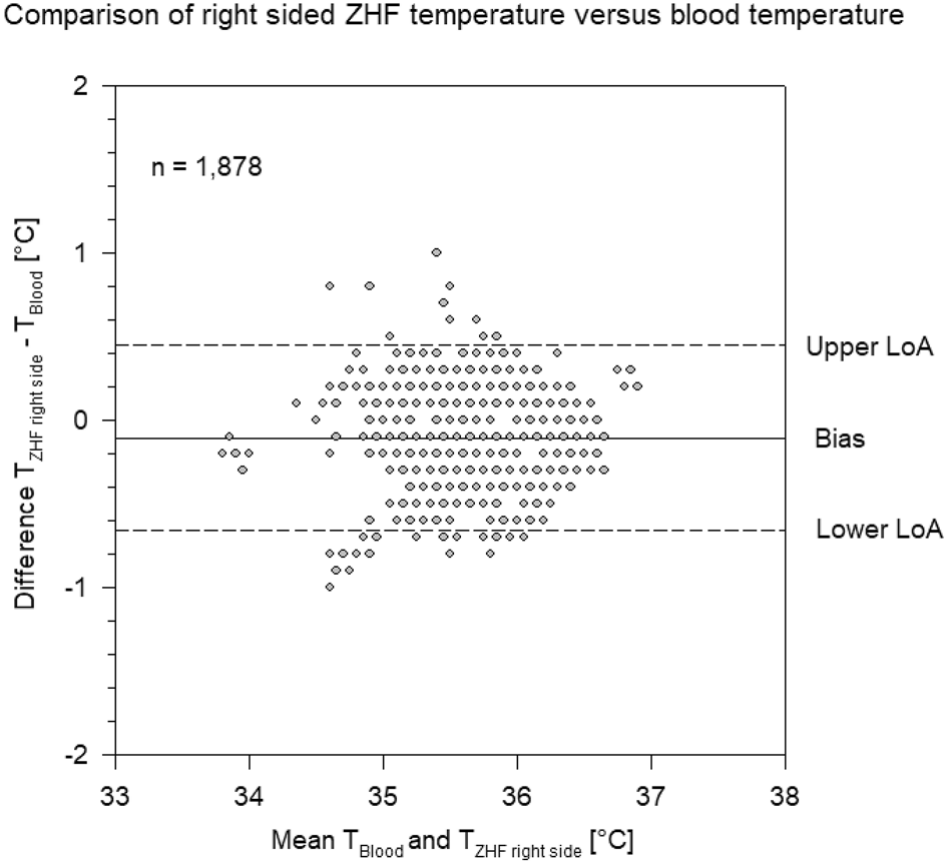
Bland–Altman plot of Zero-Heat-Flux (ZHF) temperature measured on the right side of the forehead versus blood temperature measured by pulmonary artery catheter. Each grey circle represents one or more measurement points. Bias and limits of agreement (Lo).

Bias between PAC temperature and ZHF temperature of the right side was 0.11 °C and limits of agreement (LoA) of − 0.65 °C and 0.44 °C (Fig. 1).

**Figure 2:**
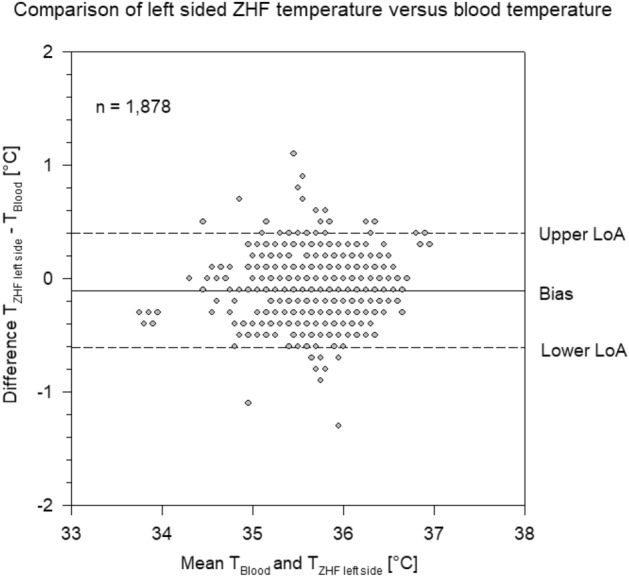
Bland–Altman plot of Zero-Heat-Flux (ZHF) temperature measured on the left side of the forehead versus blood temperature measured by pulmonary artery catheter. Each grey circle represents one or more measurement points. Bias and limits of agreement (LoA).

Bias between PAC temperature and ZHF temperature of the left side was 0.1 °C and LoA of − 0.60 °C and 0.39 °C (Fig. 2).

**Figure 3:**
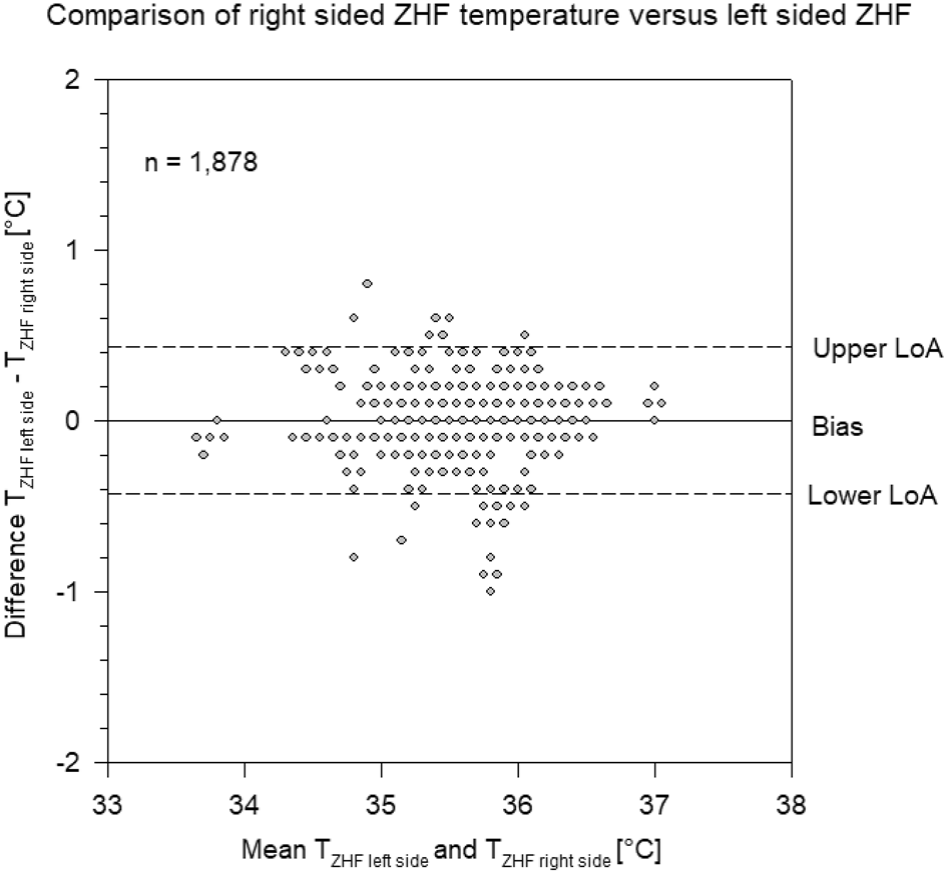
Bland–Altman plot of Zero-Heat-Flux (ZHF) temperature measured on the right side of the forehead and ZHF temperature measured on the left side of the forehead. Each grey circle represents one or more measurement points. Bias and limits of agreement (LoA).

Between the two ZHF thermometers the bias was − 0.003 °C with LoA of − 0.42 °C and 0.41 °C (Fig. 3).

**Figure 4:**
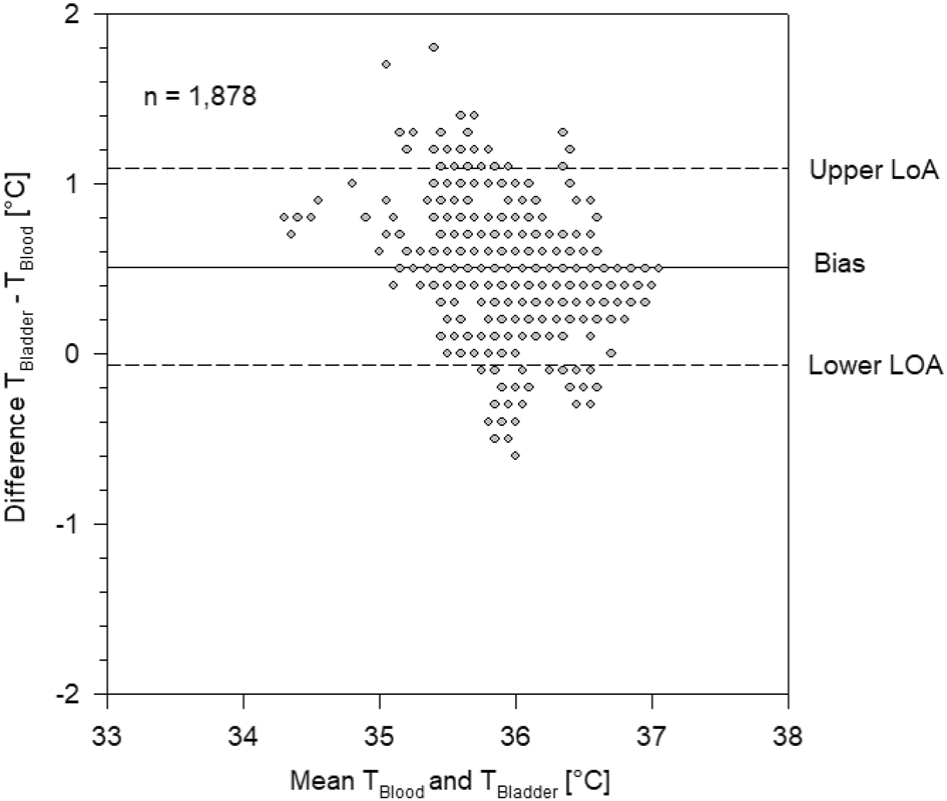
Bland–Altman plot of bladder temperature versus blood temperature measured by pulmonary artery catheter. Each grey circle represents one or more measurement points. Bias and limits of agreement (LoA).

Bias between PAC temperature and bladder temperature was 0.51 °C with LoA of − 0.06 °C and 1.1 °C (Fig. 4).

### Proportion of differences within ± 0.5 °C

In 1735/1878 (92%) of the measurements of the right sided ZHF temperatures were within ± 0.5 °C of PAC temperatures. In 1735/1878 (92%) of the measurements of the left sided ZHF temperatures were within ± 0.5 °C of PAC temperatures and 1812/1878 (96%) of the bladder temperatures were within ± 0.5 °C of PAC temperatures.

The ZHF sensors were well tolerated in all patients without occurrence of any adverse events or skin leasons. No sensor failed during the course of the measurements.

## Discussion

### Main findings

In this prospective, observational, multicentre study on 100 patients scheduled for on-pump cardiac surgery we found a high accuracy (around 0.1 °C) and a very good precision (LoA − 0.6; 0.4 °C) between the two zero-heat-flux thermometers and core temperature measured by PAC. Among the two ZHF thermometers the bias was negligible (− 0.003 °C) with narrow LoA of − 0.42 °C and 0.41 °C. In contrast, bias between bladder temperature and PAC temperature was five times larger (0.51 °C) with corresponding LoA of − 0.06 °C and 1.1 °C.

### Interpretation and comparison with relevant literature

Our results of the Bland Altman analysis of the ZHF-thermometers can be compared with four other studies that have used PAC temperature as a reference method in patients undergoing cardiac surgery. The results of Mäkinen et al. are very comparable (Bias − 0.05 °C, LoA of − 0.56 °C to + 0.47 °C), whereas the results obtained by Eshraghi et al. and Verheyden et al.^6,8^ before and after cardiopulmonary bypass show a comparable bias but a larger LoA (± 0.88 °C^6^ and ± 0.89 °C^8^). In contrast, the study of Gomez-Ramero et al.^9^ had very large LoA (− 2.27 to 2.71 °C) exceeding all other reported LoA for a ZHF thermometer in the literature. Surprisingly, the data presented graphically in the article do not match these values and LoA look smaller. Therefore, these data must be interpreted with great caution.

Our results also closely match the results other studies that used blood temperature as a comparison in the ICU for ZHF thermometers^10–12^. However, it must be stressed, that not all imprecision between the measurement sites is caused by the ZHF thermometer. Rapid infusion of cold or insufficiently warmed infusions can also lead to a rapid drop in blood temperature. In this situation, blood temperature is not representative for the temperature of the inner tissues.

To the best of our knowledge, there are no comparisons between two identical ZHF thermometers in patients yet. The agreement between the two temperature probes was very good but did not show perfect concordance. The difference between left- and right sided ZHF thermometer could represent either a device-specific variation or a physiological difference between right- and left side of the forehead, or a combination of both. Physiological differences of microvascular blood flow of the forehead has previously been shown by Benedicic et al.^13^. However, according to the theory of ZHF measurement, this should not be a relevant factor, as the ZHF device creates an isothermal tunnel under the sensor from the skin to the brain^6^. Still, it is possible, that local perfusion of the skin may influence the time till equilibrium is reached between core temperature and the skin temperature under each sensor and that this time delay is the cause of the inaccuracy.

On the other hand, these data could stress that the concept of one single core temperature is oversimplified. All studies comparing different sites of core temperature measurement show that at best, there is nearly no systematic error between the measurements, but there is always some kind of insufficient precision. For example, in ICU patients the mean bias between pulmonary artery temperature and oesophageal temperature, which is also a so-called gold standard for core temperature measurement, was 0.1 °C and the limits of agreement were ± 0.6 °C^14^.

The worse results of the Bland Altman analysis of bladder temperature versus PAC temperature are also in accordance with the literature. In general low urine flow rates reduces the accuracy of bladder temperature measurements in operative patients^15^, especially during cardiac surgery in adults and children^16,17^. Although it is well known that bladder temperature is not ideal during cardiac surgery, the measurements were included into the study because it is standard care for cardiac surgery in many centers, and at least in the ICU bladder temperature is accurate, reliable, safe and convenient^10^. Furthermore, it served as a second comparison for ZHF temperature.

### Implications

Due to the unique circumstances in cardiac surgery our results can also be of great importance in other patient groups. In our data we observe a large range of temperatures (33.6 to 37.7). This spans the range observed in most types of surgery. Even though temperatures below 34.5 °C are rare in patients undergoing non-cardiac surgery, they can be observed time and again, e.g., in in the emergency room during trauma care. However, in these situations-controlled studies to compare different core temperature methods are not possible, although it is important to obtain reliably measured core temperature values especially in these situations.

Further on, measuring the core temperatures before CPB without active warming of the patient offers the advantage to observe rapid temperature drops in a magnitude that are usual during induction and the initial maintenance of anesthesia. During the first hour of general anaesthesia the core temperature can rapidly decrease by 1 to 1.5 °C^18^.

### Strengths and weaknesses of our study

#### Strengths

This study is one of the largest studies to date that compares ZHF temperature measurements versus blood temperature measured PAC in patients undergoing cardiac surgery. In addition, it is a multicentre study, which increases the external validity. We used a Bland–Altman comparison of differences with multiple measurements to adjust for within-patient correlation. This method does not require formal rules for power calculation. Given the sample size of comparable studies^6,8,9,19^ a number of 100 patients seems to sufficiently represent the cardiac population and to demonstrate a clinically meaningful agreement between the two temperature measurement methods. The major strength is, that we were able to get temperature recordings from 100 patients who experience a fast and relatively large drop in core temperature down to core temperatures of 33.6 °C. In addition, the majority of our patients were ASA physical status classification III or IV which is known to increase the hypothermia rate significantly^20^ and enabled us to observe low core temperatures.

#### Weaknesses

In many studies comparing core temperature measurement equipment, there is a multitude of data pairs per subject and very often the number of data pairs per patient are not equal. This may induce random effects because there are influences of the different patients and influences of time in each individual patient. To account for this effect, we have used the Bland and Altman random effects method for repeated measures data adjusted for unequal numbers of measurements per patient^7^.

In our study, the gold standard of pulmonary artery temperature may have been biased by the infusion of unwarmed fluids. The magnitude of this effect depends on the temperature, the amount, and rate of the fluid given.

We also did not observe many measurements for temperatures above 37.5 °C. Therefore, it is not possible to make reliable statements about the accuracy the ZHF thermometers in hyperthermic temperature ranges from our data, although adequate accuracy for hyperthermic temperature ranges has been shown in the ICU^10,12^.

Another possible weakness of our study is that we had no mandatory screening for carotid artery stenosis in our patients. It is possible, that relevant carotid artery stenosis as well as previous stroke can reduce blood flow to the forebrain, thereby influencing forebrain temperature and ZHF thermometry. However, these theoretical possibilities have not been proven or ruled out by now.

The use of vasopressors may also have influenced the accuracy of the ZHF-thermometers. Unfortunately, we could not look at this potential source of inaccuracy in our data. This may be worthwhile to investigate in another study.

Another potential comparator for ZHF thermometry could have been nasopharyngeal temperature because nasopharyngeal temperature may better reflect cerebral temperature during cardiac surgery than bladder temperature^21^. However, nasopharyngeal temperature is not a standard in our institutions for cardiac surgery as it can lead to severe nose bleeding during CPB^22^.

Also, we did not document urine output of our patients, therefore a correlation of urine output to the accuracy of bladder temperature was impossible. However, this has already been investigated^16^.

In contrast to our study, some newer studies have used more complex statistical methods like population analysis^23^. However, in our opinion these complex analyses do not add very much new and decisive information about the accuracy of the studied devices.

We also did not investigate different risk factors such as old age, BMI > 30 kg/m^2^ and low OR temperature.

Further studies to enhance understanding of ZHF thermometers should study the influence of vasoactive substances or on the influence of external warming methods on the accuracy of ZHF thermometers.

## Conclusion

Our study shows that—in contrast to bladder temperature—ZHF thermometers are reliable core temperature monitors in cardiac surgery during the period after induction of anesthesia until CPB. The zero-heat-flux method can provide clinicians reliably with continuous and non-invasive measurements of core temperature in normothermic and mild hypothermic temperature ranges and therefore can be helpful to guide temperature management.

## Data Availability

The datasets used and analysed during the current study are available from the corresponding author on reasonable request.
